# Mutual Modulation Between Extracellular Vesicles and Mechanoenvironment in Bone Tumors

**DOI:** 10.3389/fcell.2021.789674

**Published:** 2021-12-07

**Authors:** Enrica Urciuoli, Barbara Peruzzi

**Affiliations:** Multifactorial Disease and Complex Phenotype Research Area, Bambino Gesù Children’s Hospital, IRCCS, Rome, Italy

**Keywords:** mechanoenvironment, tumor microenvironment, extracellular vesicles, bone tumors, metastatization

## Abstract

The bone microenvironment homeostasis is guaranteed by the balanced and fine regulated bone matrix remodeling process. This equilibrium can be disrupted by cancer cells developed in the bone (primary bone cancers) or deriving from other tissues (bone metastatic lesions), through a mechanism by which they interfere with bone cells activities and alter the microenvironment both biochemically and mechanically. Among the factors secreted by cancer cells and by cancer-conditioned bone cells, extracellular vesicles (EVs) are described to exert pivotal roles in the establishment and the progression of bone cancers, by conveying tumorigenic signals targeting and transforming normal cells. Doing this, EVs are also responsible in modulating the production of proteins involved in regulating matrix stiffness and/or mechanotransduction process, thereby altering the bone mechanoenvironment. In turn, bone and cancer cells respond to deregulated matrix stiffness by modifying EV production and content, fueling the vicious cycle established in tumors. Here, we summarized the relationship between EVs and the mechanoenvironment during tumoral progression, with the final aim to provide some innovative perspectives in counteracting bone cancers.

## Introduction

**FIGURE 1:**
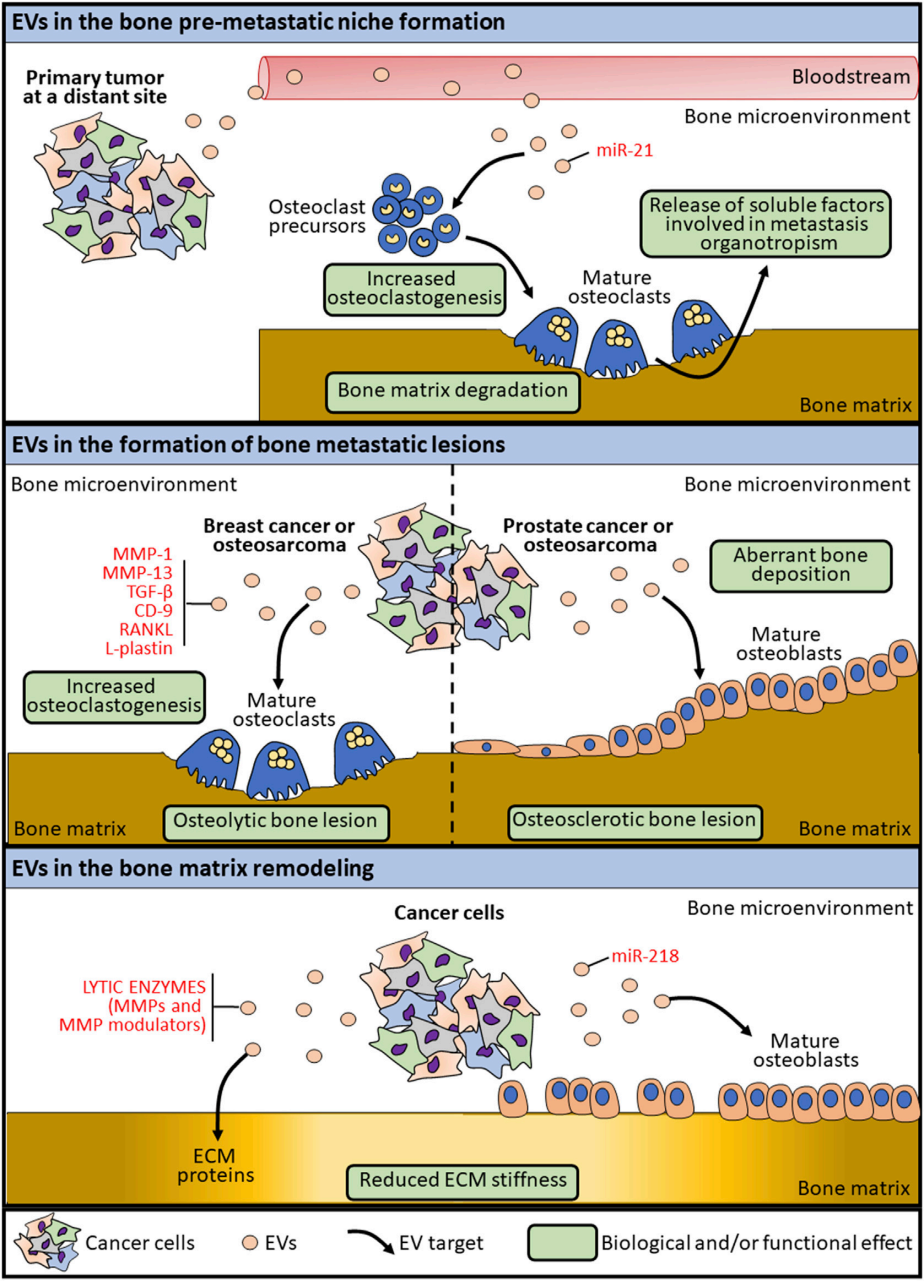
Schematic representation of the mechanisms by which EVs are involved in bone lesion formation.

Bone primary tumors are a heterogeneous group of rare neoplasms of the skeleton, accounting for approximately 0.2% of all tumors (Franchi, 2012). Their etiology is almost unknown, and an appropriate prognostication of primary bone tumors is complicated by the morphological overlap with other bone lesions of mesenchymal and non-mesenchymal origin. Bone tumors are classified as: chondrogenic, osteogenic and fibrogenic tumors, vascular tumors of bone, osteoclastic giant cell-rich tumors, notochordal tumors, other mesenchymal tumors of bone, hematopoietic neoplasms of bone and undifferentiated small round cell sarcomas of bone (Choi and Ro, 2021). Besides primary tumors, bone metastatic lesions are actually much more common, especially in adults, most of which resulting from breast and prostate primary cancers (Cecchini et al., 2005). Bone metastases are classified as osteolytic (characterized by destruction of bone matrix), osteoblastic (characterized by deposition of aberrant new bone) or mixed in dependance on how cancer cells interfere with physiological bone remodeling (Macedo et al., 2017). The main features of the metaphyseal bone, where metastases usually arise, are the rich vasculature and the constant remodeling of the bone matrix by which a plethora of soluble factors (among which growth factors, cytokines and extracellular vesicles) are released, thus acting as chemoattractant signal for cancer cells from distant sites (Bussard et al., 2008). Bone remodeling is a physiological process deriving from the continuous turnover of the bone matrix guaranteed by the finely regulated activity of the bone cells: osteoblasts (bone forming cells) (Lin et al., 2020) and osteoclasts (bone resorbing cells) (Peruzzi and Teti, 2012) that are responsible in maintaining the structural balance of bone matrix content, and the osteocytes that participate in bone remodeling in response to environmental and mechanical stimuli (Hu and Qin, 2020; Qin et al., 2020). In the bone microenvironment, the osteocytes can perceive and respond coordinately to environmental cues, such as hormones, physical stress, and mechanical loading and unloading. Doing this, osteocytes coordinate bone homeostasis by releasing factors that regulate bone formation or resorption with respect to demands (Bonewald, 2011). Several pathological factors can deregulate bone homeostasis, thereby inducing bone structural defects and macro- and micro-environment alteration, ultimately leading to cancer cells colonization within the tissue (Buenrostro et al., 2016). The mechanisms by which tumor cells metastasize to bone are poorly understood, although the theory of the premetastatic niches organized by primary tumors from near and distal regions of the body is now accepted by the scientific community (Doglioni et al., 2019; Wang et al., 2021). Once established in the bone microenvironment, cancer cells from both bone primary tumors or secondary metastases are responsible of bone remodeling deregulation, by altering bone cell activities (Taube et al., 1994; Guise et al., 2006). This process induces the release by bone cells and bone matrix of growth factors, cytokines and other soluble molecules that fuel tumor cells, leading to a “vicious cycle” in which bone integrity is destroyed and cancer cell growth and migration are promoted (Lamora et al., 2016; Esposito et al., 2018). Among the factors released by the vicious cycle, tumor- and bone-derived extracellular vesicles (EVs) are receiving growing interest because of their involvement in cancer progression and cancer bone tropism (Tamura et al., 2020) (Figure 1). EVs are non-replicable, lipid bilayer nanoscale vesicles virtually released by every cell type into the extracellular space (Yáñez-Mó et al., 2015; Théry et al., 2018). The traditional classification of EVs distinguishes three main sub-populations as microvesicles, exosomes, and apoptotic bodies (Yáñez-Mó et al., 2015; Zaborowski et al., 2015) on the basis of their biogenesis, release mechanisms, size, content and function, and their cargo consists of lipids, nuclear acids as DNAs, mRNAs, miRNAs and long non-coding RNAs, and proteins that reflect the composition of the cell of origin. Emerging evidence suggests that within a tumor microenvironment EVs are crucial factors in the communication between cancer and normal cells, influencing cancer onset, progression and metastatization (Tai et al., 2018; Dai et al., 2020). In turn, cancer cells can modulate EV production by altering the microenvironment in terms of mechanics (Eichholz et al., 2020; Nicastro et al., 2020) and acidity (Parolini et al., 2009; Logozzi et al., 2018). In the bone microenvironment, EVs are known to participate in tissue remodeling process by regulating the fine equilibrium between bone deposition and bone resorption, at both physiological and pathological levels, as well as in the tissue engineering-based bone regeneration (Eichholz et al., 2020; Yan et al., 2020). More in detail, EVs released by monocyte (Ekström et al., 2013) and osteoclasts (Wang et al., 2013; Li et al., 2016; Sun et al., 2016), as well as by mature osteoblasts (Cui et al., 2016) and osteoblast precursors (Furuta et al., 2016), are involved in regulating mesenchymal stromal cell differentiation towards osteogenic lineage. On the other side of the coin, osteoclastogenesis, the process by which bone-resorbing osteoclasts are formed starting from monocyte precursors (Zaidi et al., 2003), is mediated by bone cell-derived EVs (Cui et al., 2016; Huynh et al., 2016; Xie et al., 2017). In this context, transforming EVs derived from cancer cells are described as pivotal factors involved in bone tumor formation and progression (Han et al., 2019; Tamura et al., 2020). Indeed, EVs released by bone primary and/or secondary cancer cells are able to alter bone homeostasis favoring cancer establishment and progression (Li et al., 2019; Chicón-Bosch and Tirado, 2020; Raimondi et al., 2020). The deregulation of bone cell activities mediated by cancer-derived EVs depends on the transfer of transforming factors from cancer to normal bone cells, thus further fueling tumoral transformation and inducing aberrant bone cell activity. Most of the bone microenvironment alterations related to bone cancers are also reflected in the mechanics of the tissue, in terms of extracellular matrix stiffness and architecture. Sekita and colleagues have reported that the bone metastases induced by prostate cancer show impair mechanical functions that might be attributed to disruption of the anisotropic microstructure of bone in multiple phases (Sekita et al., 2017). Moreover, tumor-generated pressure acts to osteocytes and modifies the bone microenvironment promoting the growth of prostate cancer bone metastasis (Sottnik et al., 2015). In view of this, here we summarized the mutual relationship between extracellular vesicles and the mechanoenvironment in the context of bone tumors, both primary and metastatic, with the final aim to provide some novel points of view to counteract cancer by considering the roles exerted by EVs and the tissue biomechanics.

## Experimental Critical Issues in Studying EVs and Mechanoenvironment

From an experimental point of view, studying the extracellular vesicles is a complex challenge due to the heterogeneity of these particles (Beltraminelli et al., 2021). Furthermore, several revisions of the nomenclature referring to EVs have occurred in recent years, trying to identify a specific signature for each EV sub-populations and to standardize isolation and characterization procedures (Théry et al., 2018). The traditional classification into exosomes, microvesicles and apoptotic bodies based on biogenesis and surface protein markers can no longer be considered specific due to the overlap of size range, density and endosome-associated protein expression among EV categories. For this reason, a more recent and comprehensive classification of EVs is based on differential ultracentrifugation, the most used technique for EV isolation from cell culture media and biological fluids. By this, exosomes and microvesicles <150 nm and pelleted by 100 000g/200 000 g ultracentrifugation are collectively referred to as “small” EVs, in comparison to “large” EVs obtained by slower centrifugation (Théry et al., 2006). Therefore, given the heterogeneity of EVs and the lack of standardized techniques to isolate, purify and characterize them, it is important to keep in mind these critical issues when comparing biological results achieved by different studies. Other than any experimental bias derived by EV sample collection, purification and analysis, another level of complexity is given by the observation that EV metabolic signature depends on cell culture conditions (Palviainen et al., 2019). As deepened below, it is worth noting that the conditions in which a cell culture is maintained can modulate per se the production and the molecular signature of EVs, thus suggesting the importance in choosing the proper mechanoenvironment, in terms of both matrix stiffness and mechanical stimuli, for each cell type when the aim is studying EV production and cargo.

## Role of Tumor-Derived EVs in Regulating Bone Mechanoenvironment

Extracellular vesicles secreted by cancer cells can transform the surrounding environment as well as distant tissues, thus triggering the metastatic process, by creating favorable conditions for tumor progression (Tamura et al., 2020; Mo et al., 2021). Unlike cancers that develops in other soft organs, the establishment of a tumor lesion in the bone microenvironment implies the remodeling of the bone matrix to physically create the space for tumor growth. By a mechanism known as “the vicious cycle”, cancer cells interfere with the physiological crosstalk among bone cells leading to the bone lesion (Mundy, 2002). During this process, EVs released by cancer cells or by transformed bone cells are involved in altering the bone microenvironment and the bone matrix, thereby causing the deregulation of the stiffness and the mechanics properties of the tissue (Figure 1). The crucial processes involving EVs in bone primary tumor progression, dissemination and transformation of surrounding normal cells have been well characterized (Urciuoli et al., 2018; Chicón-Bosch and Tirado, 2020). Among these, one of the main processes by which EVs modulate the bone microenvironment is through the conveying of lytic enzymes involved in extracellular matrix remodeling, as matrix metalloproteinases (MMPs) and MMP modulators (Nawaz et al., 2018). Beside the transport of ECM-remodeling factors as cargo, osteosarcoma-derived EVs can modulate the MSC and osteoblast epigenetics by inducing the expression of MMP1 and VEGF, both involved in the bone matrix remodeling (Mannerström et al., 2019). At the same time, EVs produced by cancer cells are known to interfere with the physiological bone remodeling process, by altering the functions of both osteoblasts and osteoclasts (Figure 1). Indeed, osteosarcoma-derived extracellular membrane vesicles (EMVs) convey bioactive pro-osteoclastogenesis factors (as MMP-1 and -13, TGF-β, CD-9 and RANKL) responsible in stimulating osteoclast formation and bone resorption (Garimella et al., 2014). Raimondi et al. also described the packaging of pro-osteoclastogenesis miRNAs (miR-148a-3p and miR-21-5p) in osteosarcoma-derived exosomes that are responsible of promoting osteoclast differentiation and bone resorption activity (Raimondi et al., 2020), further demonstrating a specific role for cancer-derived exosome cargo in the alteration of bone matrix remodeling (Table 1). In the context of bone lesions induced by cancer cells developed in distant organs, EVs exploit the same pathways mentioned above to alter, directly or by transforming the surrounding cells, the bone mechanoenvironment. Prostate cancer-derived EVs are capable to affect both osteoblasts and osteoclasts, leading to osteogenic or osteolytic metastases, respectively. Exosomes collected from a prostate cancer cell line (TRAMP-C1) impair osteoclast precursor differentiation into mature osteoclasts by decreasing the expression of differentiation markers, among which cathepsin K and MMP-9 (Karlsson et al., 2016). At the same time, prostate-cancer derived exosomes overexpressing hsa-miR-940 can stimulate an aberrant osteogenic differentiation of mesenchymal cells and the activation of osteoblasts, thereby favoring the formation of an osteosclerotic lesion (Hashimoto et al., 2018) (Table 1). Breast cancer-derived EVs are known to participate in the formation of osteolytic bone lesions usually related to this type of cancer. Exosomes collected from the conditioned medium of breast cancer cells MDA-MB-231 and conveying L-Plastin are responsible in osteoclast activation, thus inducing an osteolytic bone microenvironment favoring cancer growth (Tiedemann et al., 2019). Moreover, miR-218 conveyed by MDA-MB-231-derived EVs has been described to reduced osteoblast differentiation and type 1 collagen deposition (Liu et al., 2018), thereby altering bone matrix composition (Table 1). MiRNAs conveyed by cancer cell-derived EVs can also appease the metastatic progression to bone. MiR-192 conveyed by lung adenocarcinoma-derived exosome-like vesicles is responsible alone in eliciting a multimodal mechanism by which reducing bone metastasis by acting on tumor-induced osteoclastogenesis and interfering with metastatic angiogenesis (Valencia et al., 2014) (Table 1). It is worth noting that, in the case of metastatic bone lesions, cancer-derived EVs are involved in supporting a pre-metastatic niche formation to prime successful tumor growth and survival in an otherwise hostile environment for circulating tumor cells (Guo et al., 2019) (Figure 1). Exosomes collected from conditioned medium of SCP28 cells, a bone-seeking subpopulation of MDA-MB-231 breast cancer cell line, promote the differentiation and the resorbing activity of osteoclasts, thus favoring the formation of a pre-metastatic niche *via* transferring miR-21 to bone cells (Yuan et al., 2021) (Table 1). All together, these findings demonstrate that EVs represent an important factor in the crosstalk between tumor and the host tissue and in the modulation of the bone extracellular matrix composition by interfering with bone cell activities.

**TABLE 1:** List of cited miRNAs conveyed as EV cargo and involved in the crosstalk between EVs and mechanoenvironment in bone tumors.

miRNA	Cells of origin	Target cells	Role/mechanism of action	References
miR-148a-3p	Osteosarcoma cell lines (SaOS2, MG-63, U2-OS)	Osteoclasts	To promote osteoclast differentiation and bone resorption activity	Raimondi *et al.* (2020)
miR-21-5p				
miR-940	Prostatic carcinoma cell lines (C4, C4-2 and C4-2B)	Mesenchymal stromal cells	To induce osteogenic differentiation	Hashimoto *et al.* (2018)
miR-218	Breast cancer cell line (MDA-MB-231)	Osteoblasts	To reduce osteoblast differentiation and type1 collagen deposition	Liu *et al.* (2018)
miR-192	Lung cancer cell line (A549) and highly metastatic subpopulations	Bone marrow cells and endothelial cells	To impair osteolytic lesions and bone colonization by decreasing tumor-induced osteoclastogenesis and angiogenesis *in vivo*	Valencia *et al.* (2014)
miR-21	Breast cancer cell line (SCP28)	Osteoclasts	To favor the differentiation and the resorbing activity of osteoclasts, supporting the formation of a pre-metastatic niche	Yuan *et al.* (2021)

## How Mechanical Stimuli Influence the EV-Mediated Effects in the Bone Tumor Microenvironment

The tumor microenvironment is commonly defined as desmoplastic, namely a condition characterized by chronic inflammatory status, hyperactivation of fibroblasts and pro-fibrotic pathways, elevated angiogenesis and increased production of ECM proteins responsible in stiffening the surrounding stroma, thereby altering the physiological mechanoenvironment (Pickup et al., 2014). The desmoplastic response of tissue stroma to the presence of cancer cells has been described to be driven, at least in part, by cancer-derived EVs which act on resident cells to increase ECM protein production (Jiang et al., 2017; Saber et al., 2020). In turn, matrix stiffness in the tumor microenvironment can modulate the effects mediated by cancer-derived EVs, as well as mechanical stimuli impact the EV biological properties (Piffoux et al., 2018). By using synthetic substrates mimicking the stiffness of the tumor and tumor stroma, Schwager and co-authors demonstrated that microvesicles collected from high aggressive breast cancer cell line MDA-MB-231 cultured on stiff matrix (20 kPa Polyacrylamide gels) were more able to induce a transforming phenotype in normal NIH-3t3 fibroblasts than by using softer matrices (Schwager et al., 2019). These data suggest that matrix stiffness in the tumor microenvironment can prime normal cells for response to cancer-derived EVs. Moreover, the mechanical nature of the bone matrix can influence the EV cargo. Indeed, a proteomic study on EVs produced by mineralizing vs. non-mineralizing osteoblasts revealed several differences in terms of conveyed proteins and of effects on prostate cancer cell growth (Morhayim et al., 2015), leading to the speculation that an altered mineralization status likely occurring in bone metastasis can modulate the production and the cargo of osteoblast-derived EVs. Furthermore, Morrell and co-authors showed that mechanical stimulation of osteocytes induced Ca^2+^ oscillations influencing EV release and regulating bone formation, both *in vitro* and *in vivo* experimentations (Morrell et al., 2018). Starting from these observations, a speculation can be made on an existing loop between EV cargo and release and the peculiar mechanoenvironment associated to a bone tumor lesion.

## Clinical Relevance of EV-Mechanoenvironment Crosstalk in Bone Tumors

Although the feasibility to interfere with EV production has not been already explored in the clinical approach to cancers, these vesicles are good biomarkers for cancer diagnosis and prognosis, as well as a feasible tool for drug delivery in pre-clinical and clinical applications (Ciferri et al., 2021). Similarly, as a consequence of the altered ECM processing and degradation in cancer, the protein-specific fragments deriving from ECM turnover and released in the bloodstream can be potentially used as circulating biomarkers useful for clinical diagnosis and monitoring of cancer patients (Leeming et al., 2011; Petersen et al., 2020). The relevance in considering the interplay between EV production and the bone mechanoenvironment can be instrumental in the therapeutic approaches to bone cancers. Indeed, therapies aim at reducing tissue stiffness have been exploited to decrease tumor aggressiveness and potentiate cancer treatment response. Anti-fibrotic drugs used to reduce ECM stiffening in solid cancers can also act on ECM-derived EV production by cells within the primary tumor, thereby impacting metastatization by preventing the EV-mediated conditioning of the premetastatic niche (Guo et al., 2019). Moreover, the altered ECM stiffness associated to bone tumor progression leads to changes in ECM-derived EV release, and the coordinated modulation of these two biomarkers can be monitored simultaneously, providing a new diagnostic/prognostic tool specific for the management of bone tumors. Another relevant aspect that is worth to be considered is the *in vitro* experimental conditions used in the pre-clinical studies on cancer cell culture models. The procedures historically developed for cancer cell *in vitro* experimentation involved utilizing a flat layer of cells attaching to plastic/glass substrates. Now, the scientific community has fully accepted that two-dimensional (2D) culture conditions is not physiologically relevant, and studies achieved in such conditions may difficulty be translated *in vivo* (Lovitt et al., 2014). The tissue-specific mechanoenvironment is an essential component of a tumor and must be recapitulated as much as possible in cell culture models. This can be achieved by using three-dimensional conditions and substrates with the stiffness of the tissue of origin. Notably, EV production in cell culture is strictly related to the experimental condition (Villasante et al., 2016; Palviainen et al., 2019), and this is another key aspect to be considered when cancer-derived EVs are studies in cell culture models.

## Conclusion

The tumor microenvironment is a complex structure containing cells (malignant and not), soluble factors and extracellular matrix. Cancer cells can alter the surrounding environment directly or by conditioning the normal cells, by processes involving, among others, the stiffening of the ECM and the production of transforming EVs. These two phenomena are interconnected and modulate each other, participating in a coordinated way in the onset, progression and metastasis of virtually any tumor. In the bone microenvironment, dysregulated mechanical stimuli, both in terms of matrix stiffness and of perceived mechanical forces, can act firstly on mechanosensing osteocytes, then on bone and/or cancer cells which in turn respond by releasing EVs. These can target cells in the microenvironment, interfering with the physiological bone homeostasis processes and fueling the formation and the progression of a bone tumor lesion by regulating the bone ECM composition and stiffness. Here, we recapitulated the functional crosstalk between EVs and the mechanoenvironment in bone tumors, also emphasizing the relevance to consider these aspects in the *in vitro* cell culture models for cancer drug development and personalized therapy.
